# The central role of the anesthesiologist in operating room management: toward an integrated clinical-organizational-technological paradigm

**DOI:** 10.1186/s44158-025-00263-w

**Published:** 2025-07-14

**Authors:** Valentina Bellini, Simone Priolo, Elena Bignami

**Affiliations:** 1 Anesthesiology, Critical Care and Pain Medicine Division, Department of Medicine and Surgery, University of Parma, Viale Gramsci 14, Parma, 43126 Italy; 2 Intensive Care and Anesthesia B Unit, Azienda Ospedaliera Universitaria Integrata (AOUI) Verona, Verona, Italy

## Abstract

Efficiency in the operating room is often considered either in terms of clinical excellence or in terms of performance optimization through managerial approaches. However, these dichotomous models—clinician-centered versus engineer-led—fail to capture the complexity of modern surgical care. This paper therefore proposes a multidisciplinary model in which the anesthetist plays a central role, acting as an integrator of clinical needs and organizational logistics. As new technologies emerge, they should support a comprehensive vision that combines patient-centered care with organizational and technological considerations. This approach should complement, rather than replace, clinical judgment.

To the Editor,

The management of operating room (OR) lies at the intersection of clinical precision and logistical efficiency. Traditionally, two opposing paradigms have guided OR organization: one led by clinicians with a strong focus on individualized patient care and clinical needs, and the engineer-led models with the aim to optimize throughput and performance indicators. However, this dualistic view neglects the crucial link between clinical reality and system efficiency.

According to Mahajan et al., perioperative medicine can be considered a Complex Adaptive System, composed of professionals with different roles, objectives, and norms who interact variably depending on the patient, the procedure, and the context [1]. The relationships between these agents and the hospital organization are non-linear and often influenced by institutional culture. While some standardized pathways can be represented through flows and performance indicators, most surgical pathways require flexibility, cooperation, and self-organization, as well as careful guidance aimed at promoting collaborative behaviors and reducing competition and conflict.

Among all professionals working in the OR, the anesthesiologist is uniquely positioned to bridge clinical and organizational domains. Present throughout the entire perioperative process, anesthesiologists oversee patient safety, coordinate with surgical teams, and adapt continuously to the dynamic intraoperative environment [2]. The non-technical skills typical of anesthesiology—such as stewardship, teamwork, situational awareness, risk management, and systems-level problem-solving—make anesthesiologists natural candidates for the management of elevated risk environments such as ORs [3]. Thanks to their familiarity on functional dynamics and clinical workflows across multiple medical departments, they are able to make a meaningful contribution to interdisciplinary coordination, quality assurance programs, and resource planning [2]. It is no coincidence that the investigators publishing the most in the field of OR management have been anesthesiologists [4].

It is precisely because of this bridging role that anesthetists are well placed to recognize when institutional strategies become misaligned with clinical realities. Many current OR management models—especially those built around rigid performance metrics—aim to optimize efficiency, throughput, and cost-effectiveness. However, when these models are applied without adequate clinical grounding, they risk overlooking the inherently variable and nuanced nature of patient care. The result is a system that may function well on paper but falters in practice, particularly in high-pressure environments like the OR where real-time judgment is essential.

In response to these limitations, emerging technologies such as AI and smart IoT systems are increasingly being introduced to improve operational oversight and decision-making. These tools generate valuable data-driven insights and hold promise for supporting clinical work [5]. Yet, they must not be seen as plug-and-play solutions. Their integration into perioperative workflows demands careful contextualization—both clinical and organizational—so that they enhance, rather than replace, the situational judgment that defines high-quality, safe care.

An effective and future-oriented OR management system must be multidimensional. Clinical insight, organizational strategy, and technological innovation must co-exist in a synergistic framework. In this model, the anesthesiologist is not merely a participant but a coordinator, ensuring that technological solutions and logistical protocols align with the clinical complexity of surgical care.

## Data Availability

No datasets were generated or analysed during the current study.

## Abbreviations

OR: Operating room
